# Translation, cross-cultural adaptation and psychometric properties of Urdu version of upper limb functional index; a validity and reliability study

**DOI:** 10.1186/s12891-022-05628-8

**Published:** 2022-07-20

**Authors:** Ayesha Arooj, Fareeha Amjad, Fahad Tanveer, Asad Ullah Arslan, Ashfaq Ahmad, Syed Amir Gilani

**Affiliations:** 1 University Institute of Physical Therapy, Faculty of Allied Health Sciences, The University of Lahore, Lahore, Pakistan; 2 Head of Department University Institute of Physical Therapy, Faculty of Allied Health Sciences, The University of Lahore, Lahore, Pakistan; 3 University Institute of Physical Therapy, Associate Dean Faculty of Allied Health Sciences, The University of Lahore, Lahore, Pakistan; 4 Dean Faculty of Allied Health Sciences, Directorate of International Linkages, The University of Lahore, Lahore, Pakistan

**Keywords:** Musculoskeletal disorders, Pain, Reliability, Upper limb, Validity

## Abstract

**Background:**

The upper limb functional index is broadly used outcome measure for musculoskeletal disorders of the upper limb. The main objective of the study was to translate and validate the upper limb functional index (ULFI) outcome measure in the Urdu language.

**Methods:**

Upper limb functional index was translated into Urdu language using Beaton et al. guidelines through forward and backward translation along with the expert committee reviews. Two fifty (*n* = 250) Urdu-speaking patients with sub-acute or chronic conditions of upper limb musculoskeletal disorders were included in the study. The mean age was 32.33 ± 4.67 years. The data were collected from the physical therapy department of The University of Lahore Teaching hospital. All participants completed the upper limb functional index-Urdu (ULFI-U), Numeric pain rating scale (NPRS), Quick Disability of arm, shoulder, and hand (QuickDash), and (health survey) SF-12 at baseline while only ULFI-U at day three. Reliability was assessed through internal consistency by Cronbach’s alpha and test-retest reliability by intra-class correlation (ICC). Content validity was measured by Lynn and Lawshee method. Spearman’s correlation has been used to measure criterion validity. The construct validity was measured through hypothesis testing. The structural validity has been explained through factor analysis by exploratory factor analysis (EFA) using Maximum likelihood extraction (MLE) with Promax rotation.

**Results:**

The English version of ULFI was translated into the Urdu language with minor alterations. The Urdu version ULFI has demonstrated high levels of reliability with intra-class correlation (ICC_2,1=_ 0.91) and Cronbach’s alpha (α = 0.94). The content validity index found as 0.808, the criterion validity for ULFI-U correlating with quick Dash was found excellent (r = 0.845) and ULFI-U established strong correlation with 6 domains of SF-12(r = 0.697 to 0.767) and weak correlation with its 2 domains and NPRS(r = 0.520). A two-factor structure was obtained using EFA.

**Conclusions:**

The ULFI-U is a valid and reliable patient-reported outcome (PRO) that can be used to assess upper limb musculoskeletal disorders in Urdu-speaking patients.

**Trial registration:**

This study was registered in the U. S National Library on clinicaltrial.gov under registration no. NCT05088096. (Date: 21/10/2021).

## Background

Various musculoskeletal diseases of the upper extremity are treated by health care providers in a variety of clinical settings. Following WHO’s International Classification of Functioning, health, and Disability (ICF), the participation restriction and activity limitations are important domains to compute. Therefore, therapeutic medication should aim to lessen the activity limitation caused by the disability while also improving the patient’s overall availability in societal duties [1]. Upper limb tendons, muscles, ligaments, and neural tissue are all involved in musculoskeletal problems, and the cervical spine may play a role in some cases [2]. Muscle strength, range of motion, and discomfort in performing functions are the three most important characteristics that influence function. Problems with these characteristics can induce functional loss, which can impair the activities of daily living and lead to disability. Thus, it can be a serious issue in and of itself, or it might have negative consequences on the quality of life impacted by the health of the individuals [3].

There are numerous patient-reported outcome measures (PROMs) for the assessment of the functionality of the upper extremity which is useful in health care sectors. One of the most commonly recognized PROM is the Disabilities of Arm, Shoulder and Hand (DASH) and its shortened version (QuickDASH) in different clinical and research settings [4, 5]. The DASH has different concerns such as a long administration time, takes a little longer to be filled up, dimensionality [4, 6] and responsiveness variations [7–10]. For these reasons, it has been reduced to be used in several clinical settings. Although it has a shorter version which is QuickDash, that has different concerns comparatively i.e. difference in factor structure and Rasch analysis [4]. This is the reason it has major cover points to be used as a single potential instrument [11]. Another PROM commonly recognized is Upper Extremity Functional Index (UEFI), a region-specific instrument. It has not been widely used due to its generalizability concerns because during its development, a specific working population was taken [12]. A sort of similar instrument named Upper Extremity Functional Scale (UEFS) is also known among researchers but it has shown contradictory results with its clinimetric properties such as reliability [13, 14].

A very recent PROM is the Upper limb functional index that was first formulated by C. P Gabel in the year 2006 and measured its psychometric properties [7]. The original ULFI exhibited a high level of test-retest reliability, excellent internal consistency and outstanding convergent validity when compared to other questionnaires such as Quick Dash. Before ULFI, only Dash seemed to be a reliable and valid tool for upper limb disorders. But after the formulation, ULFI appeared to be an accurate and appropriate patient-reported outcome measure (PROM) [3, 15, 16]. Moreover, ULFI has several characteristics such as is known because of its brevity, rapidity to understand, easy completion and undemanding scoring termed it as a specialized instrument for the measurement of upper limb disorders. It has been now a preferred typical regional instrument for upper limb outcome measures [7].

Pakistan is a low-income country that is still underdeveloped and is fighting to compete around the map [17]. Since there is a lot of work and less time to relax [18], professionals here are under continuous stress and heading towards different musculoskeletal disorders in which upper limb musculoskeletal disorders have also been on the list. According to Jan Hartvigsen, musculoskeletal disorders can be seen with the highest incidence in middle and lower-income countries. On the other hand, the prime concern in high-income countries is the health of an employee due to their increasing turnover. But in other countries, formal and informal factors may negatively affect the health of people in different occupations [19].

Native or/and national languages play an important part in a country’s educational development. The promotion of these languages is given a lot of significance around the world, as well as in Pakistan. In many countries, national languages are regarded as official languages. As Urdu is the national language of Pakistan therefore, it is easy for the patients to understand any question in their native language [20].

The Upper limb functional index (ULFI) is originally in the English language and most of the population, here in Pakistan, is Urdu speaking. Previously, ULFI has already been translated into several languages [3, 13, 21–25] i.e. in the indigenous languages of particular countries/regions for its better understanding there. That is why it was required to translate and cross-culturally adapt the ULFI outcome measure in the Urdu language.

The main purpose of the study was to translate the ULFI into the Urdu language and to study its psychometric properties.

## Methodology

This was a clinimetric study, conducted in a time span of one and half years, and the data were collected from March 2021 to October 2021. The study had two phases:

1. Translation and cross-cultural adaptation
2. Psychometric properties of the translated version

### Phase 1: translation and cross-cultural adaptation of ULFI

The permission to translate the original ULFI into the Urdu version was taken from the Mapi trust organization. The guidelines proposed by Gulliman and Beaton [26] were used for the translation sequence.

### Step I: forward translation

The forward translation is the initial stage of adaptation. The instrument translated from the source language i.e. English to the new target language i.e. Urdu. Two independent translators from the original language translated it into the target language. To get the best results, these two translators were having different profiles and were native Urdu speakers: a physical therapist (T1) who was aware of every term used in the instrument while the other was a professional translator (T2) and was not aware of the terms used.

### Step II: synthesis of these translations

The review committee was formed including both translators, the main author, and one physical therapist who synthesized both the translations. After discussing any alterations or dissertations, a draft of the Urdu version of ULFI was prepared. A synthesis of these translations was created using the original questionnaire, as well as the first translator’s (T1) and second translator’s (T2) versions, resulting in a single common translation (T-12).

### Step III: Back-translation

Then, the Urdu translated questionnaire was back-translated into its original language i.e. English using the T-12 version. Two bilingual translators with English as their source language created the back-translations (BT1 and BT2). These two translators had no prior knowledge of the issues being investigated i.e. no medical background. The back-translation was done to evoke unexpected meanings or to avoid any major information bias from the translated questionnaire’s items (T-12).

### Step IV: expert committee

The Expert Committee comprised of all the translators, author, and one senior physical therapist and its task were to produce the Urdu version (ULFI-U) by discussing the questionnaire’s versions and components, which included the original instrument, scoring documentation, instructions, and all the translated versions (T1, T2, T12, BT1, and BT2). Thus, the questionnaire’s pre-final version was constructed and ready for field testing.

### Step V: test of the pre-final version

The last step of the adaptation procedure was the pre-test stage. The domain test of the finalized questionnaire used the pre-final version with patients or subjects from a target context among 20 patients.

**Fig. 1:**
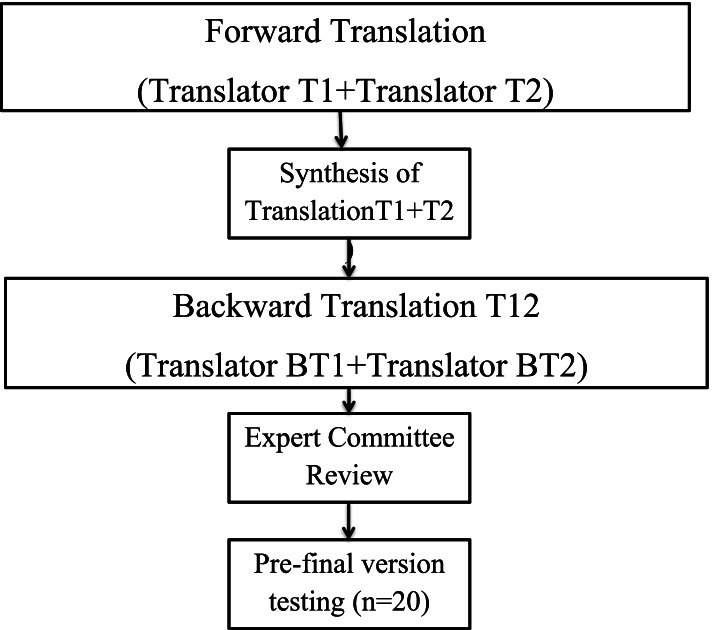
Flow chart of steps of translation and cross-cultural adaptation

Figure 1 is the flow chart for the translation steps.

### Pilot testing, cultural adaptation, face, and content validity

The ULFI-U was pilot tested on 20 patients (10 males and 10 females, mean age 30.05 ± 5.67 years) with several upper limb musculoskeletal disorders to detect any difficult wordings, alternative understandings or cultural relevance to verify the face and content validity. This was further substantiated by the expert committee discussions and reviews. Each patient was encouraged to answer the questionnaire and highlight anything that was found difficult to understand by them. Most patients found it easy to fill and understand while some minor alterations have been done before the final ULFI-U.

- ■ The Item 20 was: ‘I have difficulty eating or /using utensils (knife, fork, spoon, chopsticks), in which the word chopsticks have been removed as it is not used commonly in Pakistan.
- ■ The Item 21 was: ‘I have difficulty holding and moving dense objects (e.g.: mugs, jars, cans)’ in which teacups have been added as this is commonly used in Pakistan

After pilot testing, we invited 5 experts and provided them with the questionnaire along with a content validity questionnaire [27]. Content validity (CV) is usually assessed as content validity index (CVI) and Content validity ratio (CVR). In this study, CVI has been used because it gives results from both scale and item configuration and agreement from each expert. Moreover, this can easily be computed and only method that manages the concurrence in a single context [28, 29]. The CVI was calculated through scoring given by each member of the expert committee by a questionnaire with 1–4 items made as not relevant, somehow relevant, quite relevant, and highly relevant. Lynn and Lawshee method [30] was used to measure the content validity. The CVI has been evaluated by dividing the number of experts who gave a rating of 3 or 4 by the total number of experts. The cut-off value for CVI should be more than 0.78 [31].

### Phase II: psychometric testing

The psychometric testing of ULFI-U was made through reliability and validity testing and assessed as follows.

### Validity testing

The face, content, construct, and criterion validity were measured. The face validity was measured by interviewing patients. The content validity was measured by expert committee reviews using Lynn and Lawshee’s method [30]. The construct validity was assessed by hypothesis testing. The criterion validity was assessed by using gold standard outcome measures i.e. QuickDash and SF-12 along with NPRS. The latter two types of validity have been described further as follows:

- ■ Construct validity was measured through hypothesis testing. Hypothesis testing has been done by previous literature results. According to previously available literature [3, 13, 22, 24, 32], the priori hypothesis was made i.e. the correlation between ULFI-U and SF-12 would be weak to strong. However, ULFI-U and NPRS would be moderate while ULFI-U and QuickDash would be found strong.
- ■ Criterion validity was assessed by correlating ULFI-U with QuickDash using Spearman correlation. QuickDash was previously correlated with ULFI in many translation studies of different languages. Spearman correlation (r) was computed between ULFI-U and SF-12 and ULFI-U and NPRS. Spearman correlation has been used because ULFI is an ordinal scale. NPRS is easy to use and gives quick results for the assessment of pain. SF-12 was previously utilized in the translation of ULFI in the Brazilian Portuguese version for its reliability and validity [23]. The Spearman rank correlation(r) between − 1 to + 1 is a negative perfect correlation to a positive perfect correlation. The strength of the correlation has respective cut-off values which are 0.0–0.19 labeled as very weak, 0.2–0.39 as weak, 0.4–0.59 as moderate, 0.6–0.89 as strong, and 0.9–1.0 as very strong [33].

The structural validity was calculated using factor analysis. If the same results are not observed in the different versions of the instrument, the exploratory factor analysis should be performed to find out the latent factors [34, 35]. The EFA determines the dimensionality of the instrument. The EFA was used with Maximum likelihood extraction (MLE) through Promax rotation. There are three requirements for factor extraction i.e. a 3-priori criterion: 1) an eigen value > 1.0, variance > 10%, and the scree plot inflection at the second point [36].

### Reliability testing

The internal consistency of ULFI-U has been computed among 250 patients. Patients were descriptively introduced to ULFI-U, NPRS, SF-12, and QuickDash. The data were collected on their first visit. The internal consistency of ULFI-U was measured through Cronbach’s alpha and item-total correlation. The internal consistency assumed good when it is between 0.60–0.80 and is excellent when found between 0.80–0.95 [37].

For test-retest purposes, ULFI-U was filled by prospectively selected subgroup from the sample (*n* = 75, 32 ± 2.3 years) at their 2nd visit after 72 hours without giving them any treatment in between. The intra-class correlation (ICC_2,1_) was used to measure test-retest reliability using the two-way mixed analysis of variance [38] with a 95% of confidence interval. The ICC is considered poor, moderate, good or excellent when it is < 0.5, 0.5–0.75, 0.75–0.9 and > 0.9 respectively [39, 40]. The inter-item correlation was also computed. The alpha value for inter-item correlation > 0.7 is assumed to be good [41]. To ensure that the patient’s state changed as little as possible, the current study used a three-day gap, similar to prior studies that used fewer test-retest intervals [3, 22, 24]. A study conducted by Dawson et al. recommended a time interval of 2–3 days to avoid the changes in patient’s conditions [42].

The Bland-Altman plot was used to assess the degree of variation within-subject and the limits of agreement with a confidence interval of 95% [43]. It is used to visualize the differences between two measurements at two different time intervals [44]. For evaluating the limits of agreement of ULFI-U on two different occasions, the difference between the first and second measurements was plotted against the average of these two measurements.

Standard error of measurement (SEM) was used for the calculation of measurement error along with minimal detectable change (MDC_95_) with a confidence interval of 95% [7, 45]. The formulas such as SEM = SD × √ 1 – ICC [46] and MDC = 1.96 × √2 × SEM [47], were used to calculate.

### Participants characteristics

A total of 250 patients were recruited for the study, the sample size was computed using Kline rule of method i.e. 10:1 patients to item ratio, n = 250 [48]. The mean age calculated was 32.33 ± 4.67 years. The data collection procedure was started after the approval from the Institutional review board committee of the University of Lahore with reference no. IRB-UOL-FAHS/882/2021. All the methods were performed using proper guidelines and techniques. Before the collection of data, informed consent was duly signed by the patients enrolled and the procedure was verbally explained to them. The inclusion criteria were 18–40 years of age [22]. Both male and female gender patients were included; enrolled in a physiotherapy program for shoulder, arm/wrist, or hand musculoskeletal injury with the symptoms duration of ≤12 weeks and diagnosed by a medical practitioner [7, 21, 22]. Sub-acute or chronic upper limb musculoskeletal disorders such as tendonitis or tenosynovitis, carpal tunnel syndrome (CTS), the cramp of the hand or forearm from prolonged periods of repetitive movement, osteoarthritis or hand-arm vibration syndrome (HAVS) were included. The patients with unilateral disorders and capable of understanding and completing the self-reported questionnaires were added. The exclusion criteria were the presence of any systemic disease or severe inflammatory arthritis diagnosed with physical examination and patients with any neurological disorder. The upper limb involvement is due to any recent surgery in less than 6 weeks.

### Instruments

QuickDash, NPRS, and SF-12 were used in addition to the Urdu version of the upper limb functional index (ULFI-U).

### Upper limb functional index (ULFI)

ULFI marked as 3 points in yes scored as 1; partly scored as 0.5 and no scored as 0. Hence, ranging from 0 to 25, then the acquired score multiplied by 4 to a 100 point maximum indication of maximum disability while a 0 score shows no disability. Hence, the functional index scaled from worst function as 100 to best function as 0 can be the maximum or before injury status [9, 49].

### Disabilities of arm, shoulder, and hand (QuickDash)

The Disabilities of the Arm, Shoulder, and Hand (DASH) Questionnaire is the most commonly applied patient-reported outcome measure used to assess disability and functioning in clinical research and practice for patients with injuries and diseases of the upper extremities [50]. QuickDash is a short form of the Dash 30 items scale while quickDash has 11 items in which a missing score for one item can be accepted. It is measured on a scale of 1–100 in which the lowest score shows less disability while the highest shows more disability [51].

### Short-form health survey (SF-12)

The SF-12 **i**s a self-demonstrated outcome measure measuring the impact of health on an individual’s daily life. It is often used as a measure of the quality of life. The SF-12 is a shortened version of its original version i.e. SF-36 [52], which itself evolved from the study of Medical Outcomes [53]. It has been calculated by transforming into a range of 0–100. It has 8 domains in which 4 domains have 2 variables each while the remaining 4 domains have one variable each [54].

### Numeric pain rating scale (NPRS)

Pain is measured by a numerical Rating Scale (NRS) in which subjects are directed to describe their pain by encircling the number between 0 and 10. No pain is represented by 0–3 while the highest represents the worst pain as 7–10 and moderate pain is classified between 4 and 6 [55].

### Data analysis

SPSS version 23 was used for the data analysis procedure. The *p*-value (*p* < 0.05) was considered to be statistically significant. The obligated factor structure was measured using exploratory factor analysis in factor analysis. The verification of psychometric properties was made by formulating a-priori hypothesis.

### Priori hypothesis

According to previously available literature [3, 13, 22, 24, 32], the priori hypothesis was made. If 75% of the results would match this priori hypothesis, the validity of the scale will be considered good [56]. The priori hypothesis has been described in Table 1.

**Table 1:** Priori hypothesis for ULFI-U

Instrument	To be measured	Validity
QuickDash	Functional disability	Strong correlation (r = 0.79–0.90) [13, 21, 23–25]
SF-12	Functional disability and general health	Weak to moderate correlation (r = 0.10–0.75) [6, 7, 23]
NPRS	Pain intensity	Moderate to strong correlation (r = 0.40–0.80) [6, 7, 32]

## Results

### Translation and cross-cultural adaptation

The ULFI-U was completed by 250 patients with different upper limb musculoskeletal disorders. The participant filled in the questionnaire as it was easily applicable to their presenting complaints and has the quality of brevity. The cultural-linguistic adaptions must be kept into consideration to make sure that the new instrument is suitable for the target population [57].

Variations were then made using much finer framing the revisions which ensured all translators to agree on a final format with the following changes in comparison with the original version. None of the patient reported any difficulty while completing the ULFI-U questionnaire. Moreover, there were no missing responses found i.e. all the items received a response.

### Psychometric testing

The psychometric properties were reported as follows. Table 2 explains the demographic properties of the patients including gender, affected side involved, employment status, and region-based disorders.

**Table 2:** Demographic characteristics

Variables	Participants N (%)
**Gender**	
Male	164 (65.6)
Female	86 (34.4)
**Affected Side**	
Right	188 (75.2)
Left	62 (24.8)
**Employment status**	
Employed	208 (83.2)
Unemployed	42 (16.8)
**Pain status**	
3–6 weeks	154 (61.6)
≥ 6 weeks	96 (38.4)
**Disorders**	
Carpel tunnel syndrome	89 (35.6)
Supraspinatus tendinitis	50 (19.9)
Rotator cuff injury	46 (18.3)
Vibration syndrome	19 (7.6)
Osteoarthritis	46 (18.3)

### Reliability

**Fig. 2:**
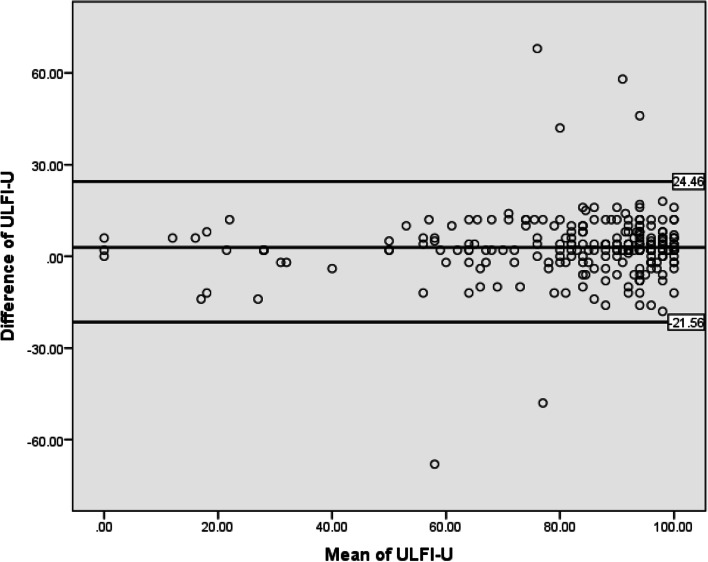
Bland and Altman plot for assessment of the limits of agreement

The ULFI-U showed excellent test-retest reliability with intra-class correlation (ICC) value of (0.91; 95% CI = 0.82–0.95). The internal consistency of ULFI-U was found excellent as the value of Cronbach’s alpha obtained was 0.94(**α** = 0.94). The item to total correlation has been measured using spearman rank correlation that depicts the strength of association between each item and overall ULFI-U minus the score of the item that is being investigated [21]. When the value is larger than 0.7, it indicates a strong association between two variables. The higher the coefficient value, the stronger the correlation between the item and the overall score, which will be ensuring that the scale is internally consistent [58]. The Item-total correlation value had ranged from 0.92 to 0.95 that is also affirming that ULFI-U is an internally consistent instrument. The Standard error of measurement (SEM) calculated was 3.89 with minimal detectable change (MDC_95_) as 10.6. The item-to total correlation and Cronbach’s alpha if item-deleted have been mentioned in Table 3. Figure 2 shows the Bland and Altman plot which is showing the variations in the subjects and limits of agreement (LOA). A small mean difference (d) = 2.9 was calculated as systematic bias and the limits of agreement ranged from − 21.56 to 24.6. The score of 4 participants was out of the limits as shown in Fig. 2. The strong agreement has been shown by the Bland and Altman plot with minimal within-subject variation between the scores of two occasions. Thus, supporting the ICCs calculated.

**Table 3:** Reliability Analysis of ULFI-U

Items	Item to total Correlation	Cronbach’s alpha if item deleted
Item 1	.648	.947
Item 2	.904	.944
Item 3	.802	.945
Item 4	.611	.947
Item 5	.494	.948
Item 6	.766	.945
Item 7	.647	.946
Item 8	.584	.947
Item 9	.655	.946
Item 10	.596	.947
Item 11	.651	.946
Item 12	.216	.951
Item 13	.608	.947
Item 14	.536	.948
Item 15	.267	.952
Item 16	.522	.948
Item 17	.547	.948
Item 18	.768	.945
Item 19	.716	.945
Item 20	.880	.944
Item 21	.918	.944
Item 22	.600	.947
Item 23	.857	.944
Item 24	.621	.947
Item 25	.898	.944

### Validity

Four types of validity i.e. face, content, construct, and criterion validity were observed. Face validity was measured by interviewing the patients. The content validity ratio for each item was in the range of 0.6–1 for each item in the scale. The content validity index for the scale was measured as 0.808.

According to Table 4 which shows the validity analysis, ULFI-U was found moderate positive when correlated with NPRS(r = 0.520) and weak to strong positive with SF-12 items(r = 0.08 to 0.767). All the items of SF-12 were found strongly positive with ULFI-U except mental health and vitality which showed a weak positive correlation and insignificant results with VIT = (r = 0.08, *p* = 0.169) and MH = (r = 0.116, *p* = 0.06). While correlating with QuickDash it showed a strong positive Spearman correlation i.e. (r = 0.845).

**Table 4:** Validity analysis of ULFI-U

Items	Correlation (r)	***p***-value (p)
**Numeric pain rating scale (NPRS)**		
**NPRS and ULFI-U**	0.520	0.001
**SF-12 Domains**		
**Physical functioning (PF) and ULFI-U**	0.767	0.001
**Role physical (RP) and ULFI-U**	0.729	0.001
**Bodily pain (BP) and ULFI-U**	0.667	0.001
**General health (GH) and ULFI-U**	0.745	0.001
**Vitality (VT) and ULFI-U**	0.087	0.169
**Sports function (SF) and ULFI-U**	0.694	0.001
**Role emotional (RE) and ULFI-U**	0.710	0.001
**Mental health (MH) and ULFI-U**	0.116	0.06
**Criterion Validity**		
**QuickDASH score and ULFI-U**	0.845	0.001

The Hypothesis has been made to measure construct validity that was observed and hence, the priori hypothesis was accepted that the correlation between ULFI-U and SF-12 found weak to strong [7, 23]. The moderate correlation was found between ULFI-U and NPRS which was evident through previous literature [7, 32]. Although QuickDash with ULFI-U was found to be strong, evident in French Canadian and the original version of ULFI [22, 32].

**Fig. 3:**
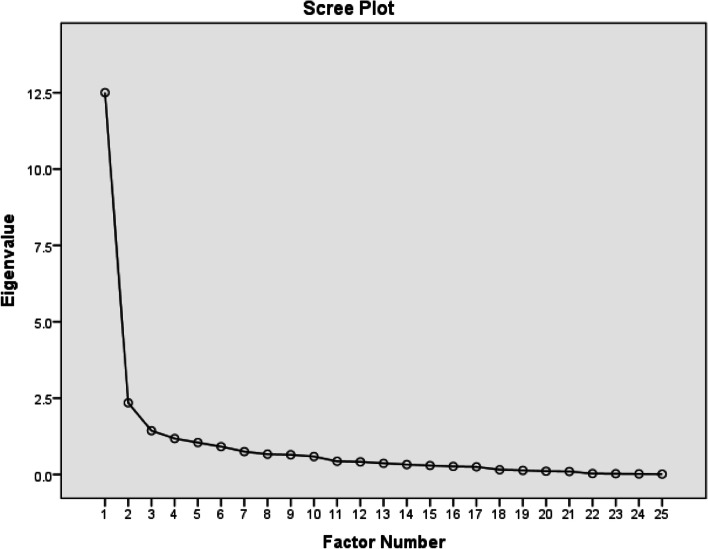
Scree plot of factor analysis for total items

The structural validity was measured by factor analysis to evaluate the factor structure of ULFI-U. The measure of sampling adequacy was calculated by the Kaiser-Meyer-Olkin (KMO) which showed that the KMO value was sufficiently high (0.928) i.e. more than 0.5 [59] and significant results (*P* < 0.001) were found with the Barlett’s test of sphericity. The extraction method of exploratory factor analysis was used. A two-factor component of ULFI-U was obtained. The Promax rotation was applied. Table 5 shows the factor loading of all items. Figure 3 explains the scree plot for factor analysis. The eigenvalue of the very first and second factor was 11.03 giving 44.1% and 5.35 giving 13.09% variance respectively. The first factor was labeled as ‘activities of daily living’ and the second factor as ‘function’. Table 6 explains the further detail of both factors.

**Table 5:** Factor loading

Items	Factors	
	1	2
Stay at home most of the time	.525	
Change positions frequently	.693	
Avoid heavy jobs	.619	
Rest more often		.658
Get others to do things	.403	
The pain almost all the time	.591	
Lifting and carrying	.512	
Appetite affected	.610	
Walking/normal recreation/sport	.568	
Home/family duties and chores	.634	
Sleep less well	.551	
Assistance with personal care, hygiene		.098
Regular daily activity work/social	.466	
More irritable/bad-tempered	.381	
Feel weaker or stiffer	.178	
Transport independence	.411	
Arm in shirt sleeve/dressing	.453	
Writing/using keyboard or mouse	.626	
Do things at/above shoulder	.683	
Eating: using utensils	.852	
Hold or move dense objects	.708	
Drop things-minor accidents		.973
Use another arm more often	.844	
Difficult button key coins taps		.979
Open, hold, press, or push	.842

**Table 6:** Psychometric properties of each factor

Psychometric properties	Factor 1	Factor 2
**Mean ± SD**	69.6 ± 19.57	13.5 ± 3.82
**Cronbach’s alpha**	0.947	0.758
**Test-retest reliability** **ICC (95% CI)**	0.89; 95% CI =0.82–0.92	0.62; 95% CI =0.58–0.74
**Correlation**	0.924	0.569

## Discussion

In this study, the ULFI questionnaire was translated into the Urdu language for better understanding across Urdu speaking population. The upper limb functional index has already been translated into different languages i.e. Brazilian Portugese [23], Italian [25], Korean [24], Persian [21], Spanish [13], French Canadian [22], and Turkish [16]. There were no missing responses as comparable findings with original English, Spanish and Turkish versions [3, 13, 32] which indicates that ULFI is an easy-to-understand outcome measure.

The cross-cultural adaptation method was derived from suggested and recognized guidelines [26]. During the cultural adaptation process fewer changes are often suggested by both expert committees and patients. These suggestions are indicative of cultural differences and routine customs between the original countries where the scale was first developed i.e. Australia and the Pakistani population. In the present study in item 20, the word chopstick has been removed as much of the population in Pakistan is not known of it; also it is not a widely used cutlery item in Pakistan. Similarly, the same change has been made in Turkish and Persian versions [3, 21]. This similarity could be due to the cultural resemblance between these countries and Pakistan. A final cultural alteration was made in item 21 in which teacups were added as this is the most common thing to use in Pakistan. A similar change was made in the Persian [21] version of ULFI in which the term ‘mugs and jars’ was removed from the same item because their population was unaware of these terms and a lack of cultural relevance as well. Also, in the Turkish version of ULFI the term ‘tea glass’ was added [3].

This study has more males (65.6%) than females (34.4%) which is comparable to previous studies such as the Persian version of the ULFI [21]. Aforementioned study had more females (60%) and fewer males (40%). In the current study mean age was 32.3 ± 4.67 years, with an age minimum of 18 years which is similar to many previous studies [3, 13, 21]. In this study, the right dominance (75.2%) and left dominance (34.4%) was observed. These results were found relevant in other studies [22] and this may be common as the right side is the dominant side in most of the population and hence get fatigued or injured mostly. Same in the French Canadian version of ULFI (ULFI-Fc), 90% of the subjects were right dominant and others were left dominant [22]. Previous literature during cross-cultural adaptation of ULFI included musculoskeletal disorders in which the MSDs for the shoulder were commonly seen [21, 22]. Similarly, in the present study shoulder injuries accounted for (56%).

This study has excellent internal consistency with a Cronbach alpha value of 0.94 which is also in the range of previous studies that is 0.75–0.99 [21–23]. Similarly, the Cronbach alpha for the Spanish version ULFI-SP was 0.94 [13], Italian version ULFI-1 was 0.90 [25], Brazilian Portuguese ULFI-Br 0.909 [23], Turkish version ULFI-Tk was 0.88 [3], Korean version ULFI-K was 0.94 [24]. Hence this showed that quite similar results have been extracted for ULFI-U. The item-total correlation for ULFI-U was found to be 0.91–0.95 which is found similar to the Persian version of ULFI (ULFI-Pr) i.e. 0.90–0.92 [21]. In contrast, the Italian version(ULFI-I) showed low item-total correlation i.e. 0.45–0.73 [25].

The test-retest reliability was calculated through intra class correlation (ICC_2, 1_), in the present study it was found excellent to be (0.91; 95% CI =0.82–0.95). Previous literature also confirms the relevancy by measuring ICC_2,1_ for Turkish version (ULFI-Tk) (0.72; 95% CI = 0.58–0.81), Korean version (ULFI-K) (0.90; 95% CI = 0.85–0.95), Italian version (ULFI-I) (0.94; 95% CI = 0.87–0.97), French Canadian version (ULFI-Fc) (0.92; 95% CI = 0.87–0.94) and Spanish version (ULFI-Sp) (0.93; 95% CI = 0.92–0.95) [3, 13, 22, 24, 25]. ICC_2,1_ of present study is similar to Spanish version of ULFI and shows neglectable changes with other versions of ULFI. The original English version ULFI showed 0.95 with 95% confidence [32].

The present study reveals the standard error of measurement (SEM) and error from minimal detectable change (MDC) as 3.89 and 10.6% respectively which is comparable to previous studies that are found as 2.94 and 5.35% [3], 3.11, and 7.25% [21], 6.11 and 14.25% [23] found in Turkish, Persian and Brazilian Portuguese versions of ULFI respectively.

Criterion validity was measured by using Spearman correlation using other patient-reported outcome measures of disability of shoulder, arm, and hand (DASH) and SF-12, and in the present study, ULFI-U showed a strong correlation with Q-Dash with a value of 0.845 and the significance < 0.001, here quickDash of 11 items was used. Only a few studies were assessed for criterion validity by using the Dash questionnaire in which study by Tonga et. Al. [3] for Turkish version ULFI showed a moderate correlation of r = 0.68, Korean version ULFI r = 0.72 [24], ULFI-Pr showed r = 0.71 and study conducted by Hamasaki et. Al. for ULFI-FC [22] and original ULFI [32] showed r = 0.85. Thus it shows that criterion validity ULFI-U is closed to the value calculated by the original ULFI and ULFI-Fc [22]. ULFI-Tk [3, 7] showed moderate correlation might be due to the sample size, etc. The ULFI-Br showed a negative high correlation with quickDash r = − 0.721 [23]. In comparison, the Spanish version of ULFI was assessed for criterion validity using Eurqol Health questionnaire 5 dimensions (EQ-5D-3L) in which inverse fair correlation was computed (r = − 0.59) [13].

Further, numeric pain rating scale (NPRS) and SF-12 were also correlated with ULFI-U in which Spearman correlation for NPRS was found moderate positive with r = 0.520 with significance < 0.001. Comparatively, the Brazilian version of ULFI showed moderate to low correlation [23]. This present study showed strong correlation with all the domains of SF-12 ranging (r = 0.697–0.767) except for the mental health (MH) and vitality (VIT) that showed weak correlation. Similarly, ULFI-Br showed moderate to low correlation with SF-36 found with all its eight domains [23].

The content validity of the ULFI-U was assessed through Lynn and Lawshee method for measuring the content validity index (CVI) which appeared to be 0.808 which is > 0.70, hence found acceptable. Only the Persian version of ULFI (ULFI-Pr) measured content validity but using the Waultz method [21, 60], relevancy within the items was found to be 0.96 which also showed excellent content validity [21].

In this study, a two-factor structure was obtained. The six-factor components with Eigenvalues > 1.0 were obtained through factor analysis, but two factors had a variance > 10% which reported for 44.1 and 13.09% of the variance in it and equated to the ‘elbow ‘of the Scree plot’ or the point of inflection. In the original English version, seven-factor components with Eigenvalues>1.0 were obtained but only one factor had shown a variance >10% (33.4%), Therefore, a one-factor structure was found. In the Spanish version (ULFI-Sp), the four factors components showed the Eigenvalues >1.0 and only one had shown >10% variance, which reported for 49% of the variance in total, in which the ‘elbow’ in the Scree plot’ was obtained at the second point. An exploratory single dimension factor structure was found in both the original English version of ULFI and the Spanish version. The English version [32] found the highest factor with the variance of 33.4% and the six other factors had shown the Eigenvalues> 1.0 while only one factor showed > 10% of the variance. Similarly in the Spanish translated version of ULFI [13] the variance of 48% was given by the dominant factor while additional three factors were found with Eigenvalues> 1.0 and only one factor was found with > 10% of the variance. Similar to our results, the Turkish version ULFI [3] study showed seven-factor values with Eigenvalues>1.0 and only two explained variance>10% which showed 18.1 and 13.1% of the total variance in both factors respectively, hence a two-factor structure was obtained. The scree plot inflection was indicated on the third point which had also confirmed a two-factor component that authenticated these results. A total of nine items in ULFI-Tk explained the Eigenvalues below 0.50 but on other hand, there were fourteen items in the original version. Likewise, The Persian version ULFI-Pr [21] explained six factors with eigen values >1.0 and only one showed variance>10 which was about 38%. The French Canadian and Korean versions did not account for factor analysis [22] and the Italian version [24] showed a multifactorial approach that might be due to the increased sample size [25].

This study can help clinicians and researchers in the future as Urdu-speaking patients may easily fill in the data and their functionality can be measured.

The strengths of the study include that the standard guidelines were used for the translation of the instrument and to measure its psychometric properties. The ULFI-U can help clinicians to communicate with the Urdu-speaking population with upper limb musculoskeletal disorders.

There could be some limitations of the study as well such as the patients found burden in completing several questionnaires at a time. This could be a field of further consideration. Another limitation is the sub-acute and chronic conditions, which may alter the functionality of the ULFI scale.

## Conclusion

It is concluded that ULFI-U is a psychometrically valid and reliable patient-reported outcome (PRO) that can be used in the assessment of the upper limb. This owns an easy and simple language that might be easily understood by patients who speak Urdu. Thus, the researchers and clinicians might use ULFI-U for the assessment of upper limb musculoskeletal disorders.

## Data Availability

All data generated or analyzed during this study are included in this published article.

## Abbreviations

ULFI: Upper limb functional index
WHO: World health organization
ICC: Intra-class correlation
SEM: Standard error of measurement
MDC: Minimal detectable change
ULFI-U: Urdu version of upper limb functional index
NPRS: Numeric pain rating scale
SF-12: 12 items short-form health survey questionnaire
MH: Mental health
VIT: Vitality
